# Intermittent hypoxia-induced protein phosphatase 2A activation reduces PC12 cell proliferation and differentiation

**DOI:** 10.1186/1423-0127-21-46

**Published:** 2014-05-16

**Authors:** Tsung-I Chen, Hung-Wen Chiu, Yi-Chung Pan, Shih-Ting Hsu, Jian-Hong Lin, Kun-Ta Yang

**Affiliations:** 1 Center of Physical Education, Office of General and Basic Education, Tzu Chi University, Hualien, Taiwan; 2 Master program, Physiology and Anatomical Medicine, School of Medicine, Tzu Chi University, Hualien, Taiwan; 3 Department of Life Science, Tzu Chi University, Hualien, Taiwan; 4 PhD program in Pharmacology and Toxicology, School of Medicine, Tzu Chi University, Hualien, Taiwan; 5 Department of Physiology, College of Medicine, Tzu Chi University, No.701, Sec. 3, Chung-Yang Rd, Hualien 97004, Taiwan

**Keywords:** Oxidative stress, Apoptosis, Cell viability, Cell cycle, Neurite outgrowth

## Abstract

**Background:**

Intermittent hypoxia (IH) plays a critical role in sleep breathing disorder-associated hippocampus impairments, including neurocognitive deficits, irreversible memory and learning impairments. IH-induced neuronal injury in the hippocampus may result from reduced precursor cell proliferation and the relative numbers of postmitotic differentiated neurons. However, the mechanisms underlying IH-induced reactive oxygen species (ROS) generation effects on cell proliferation and neuronal differentiation remain largely unknown.

**Results:**

ROS generation significantly increased after 1–4 days of IH without increased pheochromocytoma-12 (PC12) cell death, which resulted in increased protein phosphatase 2A (PP2A) mRNA and protein levels. After 3–4 days of IH, extracellular signal-regulated kinases 1/2 (ERK1/2) protein phosphorylation decreased, which could be reversed by superoxide dismutase (SOD), 1,10-phenanthroline (Phe), the PP2A phosphorylation inhibitors, okadaic acid (OKA) and cantharidin, and the ERK phosphorylation activator nicotine (*p* < 0.05). In particular, the significantly reduced cell proliferation and increased proportions of cells in the G_0_/G_1_ phase after 1–4 days of IH (*p* < 0.05), which resulted in decreased numbers of PC12 cells, could be reversed by treatment with SOD, Phe, PP2A inhibitors and an ERK activator. In addition, the numbers of nerve growth factor (NGF)-induced PC12 cells with neurite outgrowths after 3–4 days of IH were less than those after 4 days of RA, which was also reversed by SOD, Phe, PP2A inhibitors and an ERK activator.

**Conclusions:**

Our results suggest that IH-induced ROS generation increases PP2A activation and subsequently downregulates ERK1/2 activation, which results in inhibition of PC12 cell proliferation through G_0_/G_1_ phase arrest and NGF-induced neuronal differentiation.

## Background

Intermittent hypoxia (IH) may occur during intense exercise, obstructive sleep apnoea (OSA) and obstructive lung disease [1], is characterised as short cyclic episodes of hypoxia, followed by normoxia. IH is associated with increased intracellular reactive oxygen species (ROS) generation during the reoxygenation phase [2]. IH has been reported to result in partially irreversible memory and learning impairments in both animals and humans [3]. This is associated with hippocampus impairments [4,5] that result from decreases in both precursor cell proliferation and the relative numbers of postmitotic differentiated neurons [6].

ROS may play a role in inhibiting the leukemic cells proliferation [7] and the differentiation of rabbit bone marrow stromal [8] and neuroblastoma cells [9] by activating numerous signalling pathways that involve extracellular signal-regulated kinases 1/2 (ERK1/2) [10]. ERK and ERK-dependent NF-κB activation is required for oxidative stress-induced osteoblastic differentiation inhibition by primary rabbit bone marrow stromal cells and calvarial osteoblasts [8]. ERK1/2 signalling is also causally linked to the transcriptional activation of those genes required for cell proliferation and differentiation [11].

Protein serine/threonine phosphatase 2A (PP2A) controls the phosphorylation of numerous proteins involved in cell signalling [12] and has important roles in regulating cell cycle progression, apoptosis, growth, and cell-fate determination [13]. One characterised PP2A function involves regulating Ras-Raf-mitogen-activated protein (MAP) kinase signalling pathways. PP2A activation can dephosphorylate and inactivate both MAP/ERK kinase (MEK) and ERK family kinases in vitro [12,14]. In contrast, when PP2A is inhibited, phosphorylation and substrate kinase activation is induced, which further accelerates growth [13]. In the dorsal and ventral medulla of rats, the PP2A protein phosphatase expression can be regulated by IH, which is dependent on increased ROS generation [15]. However, the mechanisms for how IH-induced ROS generation affects cell proliferation and neuronal differentiation remains unknown.

Pheochromocytoma-12 (PC12) cells adopt a round morphology and proliferate to high density when cultured in serum-containing medium. PC12 cells have been widely used in both neurobiological and neurotoxicological studies as a model of neuronal differentiation [16] because they exhibit a unique sensitivity to changes in O_2_ concentration and are frequently used to study neuronal vulnerability to hypoxia [3]. Thus, we used PC12 cells to investigate the IH-induced ROS generation effects on cell proliferation and neuronal differentiation, and investigated the involvement of the ERK1/2 and PP2A signalling pathways during these processes.

## Methods

### PC12 cell culture

Rat PC12 cells were plated on dishes coated with poly-L-lysine (P1399; Sigma, USA) and cultured in Dulbecco’s modified Eagle’s medium (Gibco, USA) supplemented with 10% horse serum and 5% fetal bovine serum containing 1% penicillin and streptomycin. The cells were then grown under 21% O_2_ and 5% CO_2_ at 37°C. For experiments involving cell differentiation, PC12 cells were treated daily with 100 ng/ml nerve growth factor (NGF, 556-NG; R&D systems, USA). For experiments involving treatment using drugs (Sigma, USA) ant it concentration as follows: 10 mU superoxide dismutase (SOD), 100 μM Mn(III)tetrakis(4-benzoic acid)porphyrin (MnTBAP) chloride, 100 μM H_2_O_2_, 100 nM 1,10-phenanthroline (Phe), 2 μM okadaic acid (OKA), 10 μM U0126, 20 μM PD98059, 100 μM nicotine, and 500 nM cantharidin.

### Exposure to IH

As described in one of our previous studies [17], PC12 cells were placed in an incubator without Lucite chambers (control) or in an incubator with humidified Lucite chambers (20 cm × 20 cm × 8 cm) and exposed to normoxia (room air, RA; 20% O_2_, 5% CO_2_, and 75% N_2_) or IH (5% O_2_, 5% CO_2_, and alternating 30-min cycles of N_2_ and RA).

### Mitochondrial ROS measurements

PC12 cells were incubated with 2.5 μM MitoSOX™ Red reagent for 30 min before harvesting. After the cells were washed with phosphate-buffered saline, fluorescence was measured using the FACSCalibur Flow Cytometer (Becton Dickinson Biosciences, USA) with excitation/emission wavelengths of 510/580 nm, respectively.

### Flow cytometric analysis of cell death

Apoptosis/necrosis was determined by Annexin V-FITC Apoptosis Detection Kit (BioVision, Inc., Mountain View, CA) according to the manufacturer’s recommendations. After 4-day IH or H_2_O_2_ treatment for 2 h at 37°C, PC12 cells were washed with NT, trypsinized, harvested, and stained with Annexin V-FITC and SYTOX green (BioVision, Inc., Mountain View, CA) in binding buffer for 10 min at room temperature. Fluorescence was measured on a FACSCalibur Flow Cytometer (Becton Dickinson Biosciences, USA) The excitation/emission wavelengths for Annexin V-FITC and SYTOX were 488/530 nm, respectively.

### Real-time quantitative polymerase chain reaction (qPCR)

RNA was extracted from PC12 cells using TRIzol reagent (Invitrogen, USA), and cDNA was synthesized using the Verso™ cDNA kit (Thermo, USA). Real-time PCR was performed using the ABI 7300 Real-Time PCR system (Life Technologies, USA) with 2× Maxima SYBR green qPCR Master Mix and ROX solution (Thermo, USA). The following primer pairs were used: PP2A forward 5′-GCTCATTCTTACTGTGGCTT-3′ and reverse 5′-CGTAACATAGTCCCCCATTA-3′ and GAPDH forward 5′-ATGTTCCAGTATGACTCCACTCACG-3′ and reverse 5′-GAAGACACCAGTAGACTCCACGACA-3′. Total RNA (3 μg) was used to perform the reverse transcription reaction. A 1:10 dilution of the synthesized cDNA with RNase-free water (total volume = 25 μl) was subsequently used for qPCR. The comparative C_t_ method (2^-ΔΔCt^) was used to quantify gene expression, where ΔΔC_t_ = ΔC_t_ (sample) - ΔC_t_ (reference).

### Western blotting

PC12 cells (1 × 10^5^ cells/ml) were lysed by sonication on ice with 100 μl RIPA lysis buffer (cat. no. 20–188; Millipore, USA) containing 1% protease inhibitor (Calbiochem, USA). The cells were then centrifuged at 10,600 × *g* at 4°C for 10 min. Protein concentration in supernatants was quantified using the BSA Protein Assay kit (Biorad, USA). Proteins (30 μg/lane) were resolved on sodium dodecyl sulfate–polyacrylamide gel using the Bis–Tris Electrophoresis System (Bio-Ray, USA). Resolved proteins were then transferred to polyvinylidene fluoride membranes (Millipore, USA); the membranes were blocked with 5% non-fat milk for 1 h at room temperature and probed with dilutions of primary antibodies against β-actin (1:10000, MAB1501; Millipore, USA), ERK1/2 (1:1000, SC-94), p-ERK 1/2 (1:100, SC-7383), and PP2A (1:1000, SC-9070; Santa Cruz Biotechnology, USA) at 4°C overnight. The membranes were then incubated with the secondary antibody, i.e., goat anti-rabbit IgG or anti-mouse IgG (1:5000; Millipore, USA) labeled with horseradish peroxidase for 1 h at room temperature. The membranes were subsequently washed. All proteins were detected using the RPN2232 ECL™ Prime Western Blotting Detection Reagent (GE Healthcare, USA) and X-ray films (GE Healthcare, USA). The resulting bands were quantified as arbitrary units (OD × band area) using the Image J analysis software (National Institutes of Health, Bethesda, MD, USA).

### Immunocytofluorescent staining

Cells were fixed with methanol at room temperature (RT) for 10 min. After a 5-min incubation in 5% non-fat milk, the cells were exposed to a primary antibody against ERK for 1 h at 37°C, followed by the secondary antibody, i.e., FITC-conjugated goat anti-rabbit IgG or anti-mouse IgG (Millipore, USA), for 1 h at 37°C. Images were obtained by confocal microscopy (TCS SP2 AOBS; Leica, Germany). Nuclei of PC12 cells were stained with 2 μM Hoechst 33342 (Sigma, USA) for 15 min; the dye was subsequently rinsed out.

### 3-(4,5-Dimethylthiazol-2-yl)-2,5-diphenyltetrazolium bromide (MTT) assay

MTT was added to each dish (1:9, v/v), and cells were incubated for 2 h at 37°C until a purple precipitate was visible. The medium was then carefully removed, and the precipitate was lysed using 1 ml dimethyl sulfoxide (DMSO) with gentle shaking at room temperature in dark for 10 min. The plates were read using an ELISA plate reader (Multiskan EX, Thermo, USA) at a wavelength of 570 nm.

### Cell cycle analysis

Cells were incubated for 1 h at 4°C in 1 ml hypotonic solution containing 20 μg/ml propidium iodide (PI), 0.1% sodium citrate, 0.1% Triton X-100, and 0.2 mg/mL DNase-free RNaseA. Cells were then subjected to flow cytometric analysis, and DNA content was determined using the FACSCalibur Flow Cytometer (Becton Dickinson Biosciences, USA). This method allows for calculation of the percentage of cells in the G_0_/G_1_ (resting phase) phase, S (DNA synthesis) phase, G_2_M phase, and sub-G_1_ phase (apoptotic cells) [18].

### 5-bromo-2-deoxyuridine (BrdU) assay for DNA replication and cell division

BrdU is a synthetic thymidine analogue that becomes incorporated into newly synthesised DNA that provides a test for DNA replication and is an indirect measure of cell division. Cell proliferation was assessed using a BrdU cell proliferation ELSIA assay kit (cat. no. 2750, Millipore, USA). After removing the labelling medium, cells were fixed and DNA was denatured using a fixing solution. A mouse monoclonal antibody was used to detect BrdU in a sample. After adding a goat anti-mouse IgG-peroxidase conjugated secondary antibody, signals were measured with a spectrophotometric microplate reader (Thermo Scientific Multiskan EX) at a wavelength of 450 nm.

### Statistics

Statistical analyses were performed using the SPSS 13.0 software (SPSS, Inc., Chicago, IL, USA). All values are expressed as means ± standard errors of the means (SEM). Statistical differences were compared using the t-test and one-way analysis of variance (ANOVA) with post-hoc test; *p* < 0.05 was indicative of statistical significance.

## Results

### IH-induced mitochondrial ROS generation does not result in PC12 cell death

**Figure 1:**
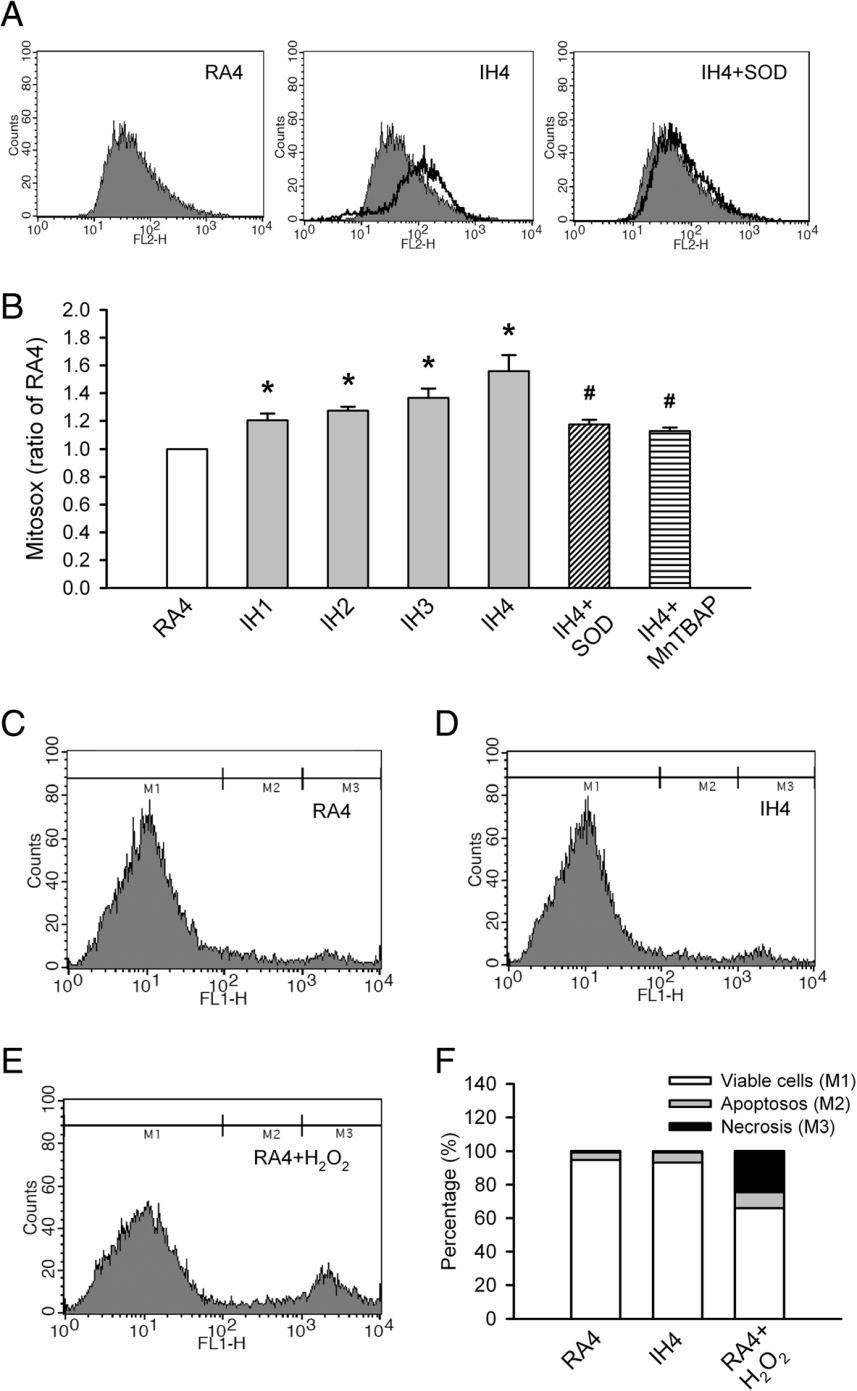
**Intermittent hypoxia (IH) effects on mitochondrial reactive oxygen species (ROS) generation and cell death in PC12 cells. (A)** Mitochondrial ROS generation was determined by flow cytometry using MitoSOX Red. **(B)** Quantitative levels of mitochondrial ROS generation in PC12 cells exposed to normoxia for 4 days (RA4, n = 16), IH for 1–4 days (IH1, n = 6; IH2, n = 6; IH3, n = 7; IH4, n = 7) and IH4 along with the superoxide dismutase (IH4 + SOD, n = 6) and Mn(III)tetrakis(4-benzoic acid)porphyrin (IH4 + MnTBAP, n = 6). **(C-E)** Percentages of viable cells (M1), apoptotic fractions (M2) and necrotic fractions (M3) were assessed using an Annexin V assay and flow cytomery. **(F)** Quantitative levels of viable, apoptotic and necrotic fractions among PC12 cells exposed to RA4 (n = 10), IH4 (n = 10) and RA4 along with H_2_O_2_ (RA4 + H_2_O_2_, n = 4). *p < 0.05 compared with RA4. ^#^*p* < 0.05 compared with IH4. Values are means ± SEMs.

Mitochondrial ROS generation, as determined by flow cytometry using MitoSOX, significantly increased after 1–4 days of IH compared with that after 4 days of RA (RA4; *p* < 0.05). To further clarify whether the increased mitochondrial ROS levels were induced by IH, SOD was added each day to the culture medium for 4 days (IH4 + SOD). Significantly lower levels of mitochondrial ROS generation were found with IH4 + SOD as compared with IH4 (*p* < 0.05). SOD was also replaced with MnTBAP, an SOD mimic as a superoxide scavenger, to confirm the IH-induced increased levels of mitochondrial ROS in PC12 cells. Similar to adding SOD to IH4 conditions, MnTBAP (IH4 + MnTBAP) abolished mitochondrial ROS generation in 4-day IH-exposed PC12 cells (*p* < 0.05; Figure 1A and 1B). To assess whether the increased mitochondrial ROS levels caused IH-exposed PC12 cells’ death, an Annexin V assay was used to determine the percentages of viable cells (M1), apoptotic fractions (M2) and necrotic fractions (M3). The percentages of necrotic and apoptotic cells did not differ between RA4 and IH4 (*p* > 0.05; Figure 1C-F). However, adding H_2_O_2_ to RA4, decreased the number of viable cells and increased the number of necrotic and apoptotic cells as compared with RA4 to IH4 alone (*p* < 0.05). These results suggested that 4 days of IH induced increased mitochondrial ROS generation but did not cause PC12 cell death.

### IH-induced ROS generation induces PP2A expression

**Figure 2:**
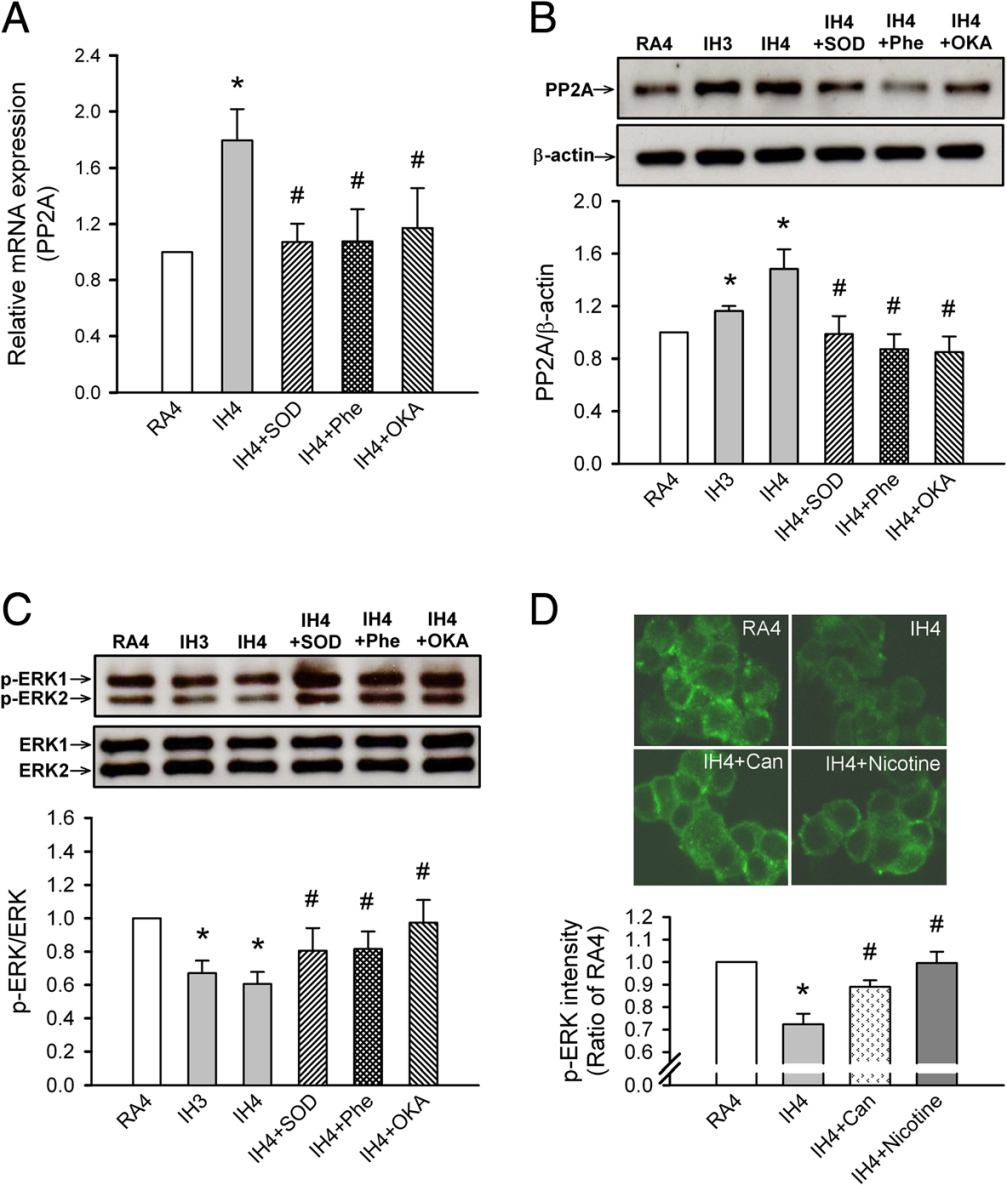
**Intermittent hypoxia (IH) effects on protein phosphatase 2A (PP2A) and extracellular signal-regulated kinase 1/2 (ERK1/2) phosphorylation. (A)** PP2A mRNA levels were determined by real-time quantitative polymerase chain reaction (qPCR) and **(B)** PP2A protein levels were determined by Western blot in PC12 cells after 4 days of room air exposure (RA4, n = 7), 3–4 days of IH (IH3, n = 5; IH4, n = 5), and IH4 along with superoxide dismutase (IH4 + SOD, n = 6), 1,10-phenanthroline (IH4 + Phe, n = 5) or okadaic acid (IH4 + OKA, n = 5). **(C)** ERK1/2 phosphorylation levels in PC12 cells were determined by Western blotting after RA4, IH3, IH4, IH4 + SOD, IH4 + Phe, IH4 + OKA and **(D)** immunocytofluorescence (green) after RA4, IH4, IH4 along with cantharidin (IH4 + Can, n = 5) and nicotine (IH4 + Nicotine, n = 5). *p < 0.05 compared with RA4. ^#^*p* < 0.05 compared with IH4. Values are means ± SEMs.

PP2A mRNA and protein expression levels were significantly upregulated after 4 days of IH (IH4) as compared with RA4 (*p* < 0.05; Figure 2A and 2B). These increased expression levels were abolished when the following were added daily to the culture medium: SOD (IH + SOD), a superoxide scavenger; Phe (IH + Phe), a Fe^2+^ chelator that reduces ROS production by inhibiting Fenton reactions and OKA (IH + OKA), an inhibitor of PP2A activation (*p* < 0.05).

### IH-induced PP2A expression attenuates ERK1/2 activation

ERK1/2 phosphorylation levels were significantly attenuated after 3–4 days of IH as compared with after RA4. This effect was abolished by SOD (IH + SOD), Phe (IH + Phe) and OKA (IH + OKA) (*p* < 0.05; Figure 2C). Moreover, immunocytofluorescent staining results showed that the levels of phosphorylated ERK (green) were significantly decreased after 3–4 days of IH as compared with after RA4 (both *p* < 0.05). This effect was abolished by cantharidin (IH4 + Can), an inhibitor of PP2A; nicotine (IH4 + Nicotine), an activator of ERK1/2 phosphorylation and OKA (IH4 + OKA) (all *p* < 0.05). However, as compared with exposure to RA4, RA4 cells treated with U0126 (IH4 + U0126), an inhibitor of ERK1/2 phosphorylation, had reduced phosphorylated ERK levels (*p* < 0.05; Figure 2D).

### IH-induced decreases in PC12 cell numbers is caused by G_0_/G_1_ phase arrest

**Figure 3:**
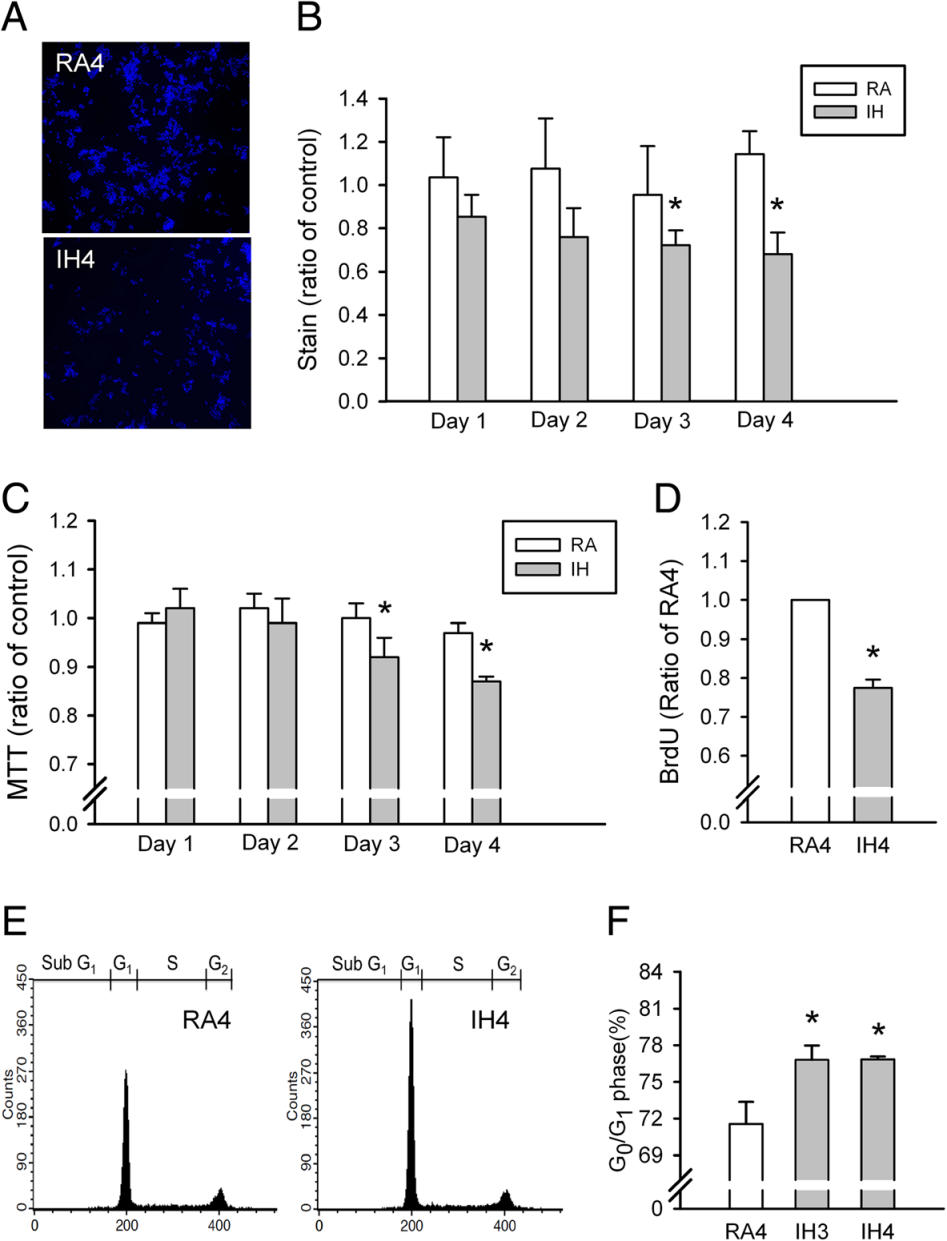
**Intermittent hypoxia (IH) effects on PC12 cell numbers, cell viability, cell proliferation and cell cycle progression. (A)** Numbers of PC12 cells as evaluated using Hoechst staining (blue) and confocal microscopy. **(B)** Quantitative PC12 cell numbers after exposure to normoxia (RA) and IH for 1–4 days (n = 5 for per group). **(C)** PC12 cell numbers as determined by MTT assay after exposure to RA and IH for 1–4 days (n = 5 for per group). **(D)** PC12 cell proliferation determined by BrdU cell proliferation ELISA assay kit after exposure to 4 days of RA (RA4, n = 8) and IH (IH4, n = 8). **(E)** PC12 cell cycle progression after exposure to RA4 (n = 8), IH3 (n = 7) and IH4 (n = 7) as evaluated by propidium iodide staining and flow cytometry. Percentages of cells in G_0_/G_1_ phase arrest (E and F). *p < 0.05 compared with RA in **(B)** and **(C)** or RA4 in **(D)** and **(F)**. Values are means ± SEMs.

The numbers of PC12 cells stained with Hoechst 33342 were not different after exposure to IH or RA on days 1–2. However, the number of these cells was significantly lower after exposure to IH than to RA on days 3–4 (both *p* < 0.05; Figure 3A and 3B). MTT assay results were reduced after exposure to IH as compared with RA on days 3–4 (both *p* < 0.05; Figure 3C). However, MTT assay results may represent a loss of cell viability or proliferation. We used a BrdU assay to confirm whether PC12 cell proliferation was inhibited after exposure to IH for 4 days. Cell proliferation was significantly reduced after exposure to IH4 as compared with RA4 (*p* < 0.05; Figure 3D). Furthermore, flow cytometry results showed that cell proliferation, as represented by the G_0_/G_1_ population, was significantly increased after exposure to IH as compared with RA on days 3–4 (both *p* < 0.05; Figure 3E and 3F).

### IH-induced ROS generation induces PP2A activation and downregulates ERK1/2 activation, thereby inhibiting cell proliferation

**Figure 4:**
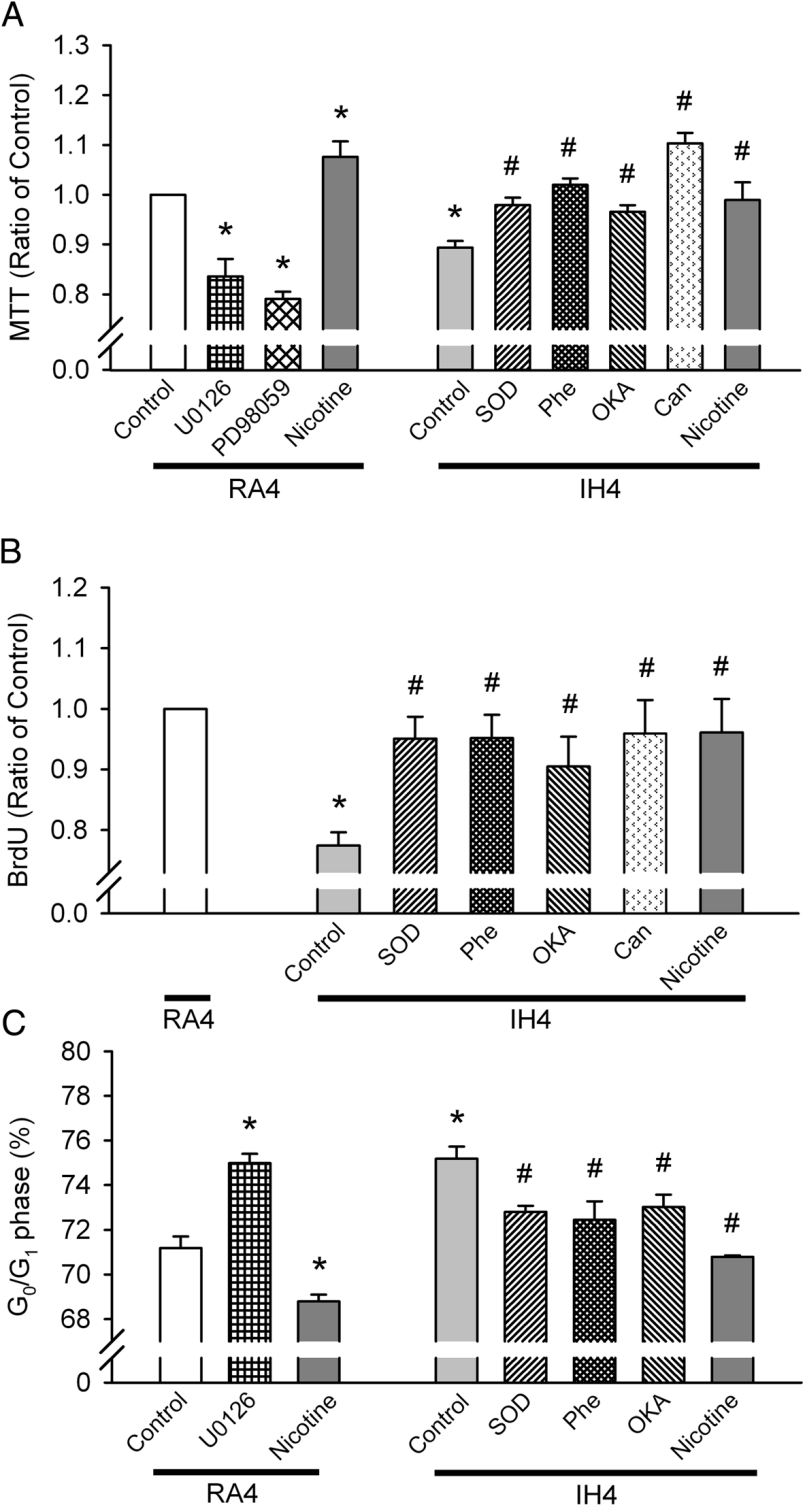
**Effect of intermittent hypoxia (IH)-induced ROS generation on PP2A activation and downregulated ERK1/2 activation leading to PC12 cell proliferation inhibition. (A)** PC12 cell numbers determined by MTT assay after exposure to normoxia for 4 days (RA4, control, n = 16), and RA4 along with the ERK1/2 phosphorylation inhibitors U0126 (n = 8) and PD98059 (n = 7), the ERK1/2 phosphorylation activator nicotine (n = 14), and exposed to IH for 4 days (IH4, control, n = 8), IH4 along with superoxide dismutase (SOD, n = 12), the PP2A activation inhibitors 1,10-phenanthroline (Phe, n = 12), okadaic acid (OKA, n = 9) and cantharidin (Can, n = 9) and nicotine (n = 9). **(B)** PC12 cell proliferation determined by BrdU cell proliferation ELISA assay kit after exposure to RA4 (n = 8), IH4 (control, n = 8) and IH4 along with SOD (n = 7), Phe (n = 7), OKA (n = 7), Can (n = 7) and nicotine (n = 6). **(C)** Percentages of PC12 cells in G_0_/G_1_ phase arrest as determined by propidium iodide staining and flow cytometry after exposure to RA4 (control, n = 8), RA4 along with U0126 (n = 4), RA4 with nicotine (n = 4), IH4 (control, n = 7), IH4 along with SOD (n = 6), Phe (n = 5), OKA (n = 5) and nicotine (n = 4). *p < 0.05 compared with RA4. ^#^*p* < 0.05 compared with IH4. Values are means ± SEMs.

As compared with exposure to RA4, RA4 cells treated with the ERK1/2 phosphorylation inhibitors U0126 (RA4 + U0126) and PD98059 (RA4 + PD98059) had reduced cell proliferation as assessed by the MTT assay, represented as the percentage inhibition of cell numbers (Figure 4A). In contrast, cell proliferation by MTT assay was significantly greater for RA4 cells treated with the activator of ERK1/2 phosphorylation nicotine (RA4 + Nicotine; all *p* < 0.05; Figure 4A). However, cell number and proliferation were significantly reduced after exposure to IH as compared with RA on day 4, which effect was abolished by SOD (IH4 + SOD), Phe (IH4 + Phe), OKA (IH4 + OKA), cantharidin (IH4 + Can), a selective inhibitor of PP2A and nicotine (IH4 + Nicotine) (all *p* < 0.05, Figure 4A and 4B). To further confirm the IH effect on cell cycle progression, the proportions of cells in the G_0_/G_1_ phase were assessed by flow cytometry. As compared with exposure to RA4, RA4 cells treated with the ERK1/2 phosphorylation inhibitor U0126 (RA4 + U0126) had increased proportions of cells in the G_0_/G_1_ phase, which reflected G_0_/G_1_ arrest (*p* < 0.05; Figure 4C). In contrast, the proportion of cells in the G_0_/G_1_ phase were significantly lower in RA4 cells treated with nicotine (RA4 + Nicotine; *p* < 0.05; Figure 4C). However, the proportions of cells in the G_0_/G_1_ phase were significantly higher after exposure to IH as compared with RA on day 4; this effect was abolished by SOD (IH4 + SOD), Phe (IH4 + Phe), OKA (IH4 + OKA) and nicotine (IH4 + Nicotine) (all *p* < 0.05; Figure 4C).

### IH inhibits NGF-induced neuronal differentiation in PC12 cells

**Figure 5:**
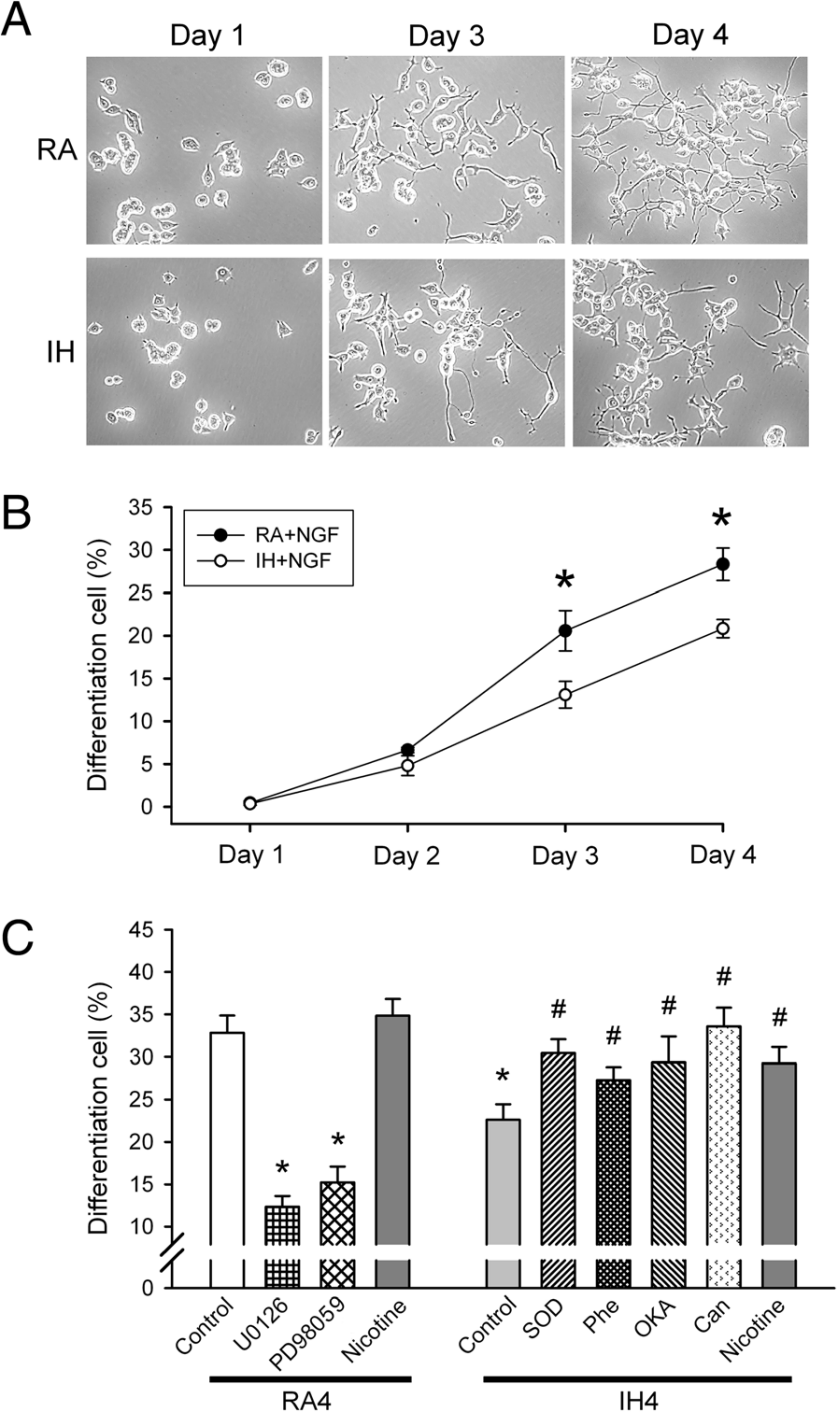
**Effects of intermittent hypoxia (IH)-induced ROS generation on PP2A activation and downregulated ERK1/2 activation leading cell differentiation inhibition by nerve growth factor (NGF)-stimulated PC12 cells. (A)** Images of neurite outgrowth using light microscopy and **(B)** percentages of differentiated cells among NGF-stimulated PC12 cells after 3–4 days of intermittent hypoxia (n = 5 for per group). **(C)** Percentages of differentiated cells among NGF-stimulated PC12 cells after exposure to normoxia for 4 days (RA4, control, n = 16), RA4 along with U0126 (n = 6) and PD98059 (n = 5) (ERK1/2 phosphorylation inhibitors), and nicotine (n = 10) (ERK1/2 phosphorylation inducer), and exposed to IH for 4 days (IH4, control, n = 14), IH4 along with superoxide dismutase (SOD, n = 8), 1,10-phenanthroline (Phe, n = 9), okadaic acid (OKA, n = 12) and cantharidin (Can, n = 5) (PP2A activation inhibitors), and nicotine (n = 7). *p < 0.05 compared with RA4. ^#^*p* < 0.05 compared with IH4. Values are means ± SEMs.

As compared with day 1, the proportion of cells with neurite outgrowths (characterised as neurites twice the cell body length) in NGF-stimulated differentiated PC12 cells was increased after exposure to RA on days 3–4. However, the number of NGF-stimulated PC12 cells with neurite outgrowths after exposure to IH was less than that after exposure to RA on days 3–4 (both *p* < 0.05; Figure 5A and 5B).

### IH-induced ROS generation induces PP2A activation and downregulates ERK1/2 activation and inhibits NGF-stimulated PC12 cell differentiation

As compared with exposure to RA4, RA4 cells that were treated with the ERK1/2 phosphorylation inhibitors U0126 (RA4 + U0126) and PD98059 (RA4 + PD98059) had reduced percentages of differentiated cells (*p* < 0.05; Figure 5C). The percentage of differentiated cells increased among RA4 cells that were treated with nicotine (RA4 + Nicotine), although this was not statistically significant (*p* > 0.05; Figure 5C). However, the percentages of differentiated cells were significantly lower after exposure to IH as compared with RA on day 4; this effect was abolished by SOD (IH4 + SOD), Phe (IH4 + Phe), OKA (IH4 + OKA), cantharidin (IH4 + Can) and nicotine (IH4 + Nicotine) (all *p* < 0.05; Figure 5C).

## Discussion

One of the major findings of this study was that mitochondrial ROS generation was increased in PC12 cells after exposure to IH and contributed to increased PP2A expression. PP2A subsequently suppressed ERK1/2 phosphorylation, which resulted in inhibiting PC12 cell proliferation through G_0_/G_1_ phase arrest and NGF-induced neuronal differentiation.

IH-induced increased cellular oxidative stress levels [17,19] can result in cell death [20,21]. In this study, although 1–4 days of IH increased the levels of mitochondrial ROS in PC12 cells, a finding consistent with that previously reported [3], an unexpected finding was that IH4 did not increase the percentages of the necrotic and apoptotic cells. This contradicted the results of previous studies that exposure to IH induced cell loss through PC12 [3] and rat primary cerebellar granule cells apoptosis [17]. This was probably because, in this study, approximately 9% of the lowest dissolved O_2_ concentration in the culture medium during hypoxia induced by IH (30 min of 5% O_2_, 30 min of 21% O_2_) was higher than the 5% O_2_ induced by other IH profiles (35 min of 5% O_2_ and 25 min of 21% O_2_) [3]. However, we previously reported that the mechanism of IH-induced apoptosis was different between cerebellar granule and other cells [17]. The IH profiles adopted by us, which induced cell loss through apoptosis of rat cerebellar granule cells but not of PC12 cells, may be related to using different cell types.

It has been suggested that H_2_O_2_-induced ROS increases PP2A expression levels in PC12 cells [22] and neurons [23]. In this study, IH-induced ROS increased PP2A expression levels, which was abolished by SOD and Phe treatments. Moreover, increased PP2A activation occurred concomitantly with decreased ERK activation in IH-exposed PC12 cells, which was similar to previous findings that ERK activation was negatively regulated by PP2A [14,24]. Because OKA not only inhibits PP2A phosphorylation but also inhibits other phosphatases, including PP1, PP4, PP5 and PP6 [12], we also used another potent, selective PP2A inhibitor, cantharidin [13], to further assess the role of PP2A in regulating ERK activation during IH, which inhibits ERK activation [25]. Our results showed that IH-attenuated ERK1/2 activation was reversed by OKA and Can in PC12 cells, which suggested that IH-induced oxidative stress increased PP2A expression and subsequently inhibited ERK1/2 activation.

Our findings disagreed with those in a previous report that rats exposed to IH (15 s, 5% O_2_; 5 min, 21% O_2_, IH15s) for 10 days had downregulation of PP2A and the upregulation of protein kinases, including PKA, CaMKII and ERK1/2 in the brainstem medullary regions. However, in contrast to IH15s, PP2A activity and the levels of active PKA and CaMKII were not affected by IH (90 s, 10% O_2_; 5 min, 21% O_2_, IH90s). In addition, the phospho-ERK1/2 level was also not affected [15]. Thus, PP2A activation regulated by IH may depend on the duration of hypoxia and the oxygen fraction in the IH pattern.

In general, low to moderate ROS can induce MAPK pathways that leads to cell growth and proliferation, whereas high ROS induce DNA damage and/or MAPK pathways that activate p53, cell arrest, and apoptosis [26]. In this study, 3–4 days of IH decreased the numbers of PC12 cells, which was consistent with a previous report [3]. Moreover, we showed that IH-induced increased ROS generation without increased cell death could induce cell-cycle arrest in the G_0_/G_1_ phase. This was probably because the cell cycle can be arrested in response to ROS and/or reactive nitrogen species which result in delayed progression through G_1_ and S phase. For example, peroxides inhibit cyclin E/cyclin-dependent kinase 2 function and the related S phase entry in a dose-dependent manner and induce a G_1_ checkpoint through the inhibition of cyclin E/cyclin-dependent kinase 2 activity [26].

In addition, an important feature of PC12 cells is that they respond to NGF with a dramatic change in their phenotype and acquire several properties characteristic of sympathetic neurons. NGF-treated PC12 cells cease to proliferate, or extend neurites and become electrically excitable [16]. Neurite outgrowth is an important aspect of neuronal plasticity and regeneration in neuropathological conditions and neural injury [27]. Consistent with previous reports [28,29], in this study, more than 20% of NGF-stimulated PC12 cells had neurites that were twice the cell body length after exposure to RA4. Although the effects of IH on neuronal cell differentiation remain poorly understood, a previous study reported that 10 cycles of IH (1% O_2_ for 24 h, following a 24-h recovery period under RA) suppressed retinoic acid-induced differentiation of neuroblastoma cells [9]. Notably, activating ERK signalling pathways reportedly regulates neuronal differentiation and protects neurons from drug-induced injury. In addition, ERK activation is required for NGF-stimulated neurite outgrowth in PC12 cells [27]. In this study, the percentage of differentiated cells with RA4 was reduced by two inhibitors of ERK phosphorylation, U0126 and PD98059, which was consistent with previous reports [16,30,31]. In contrast, the percentage of differentiated cells was increased by an activator of ERK phosphorylation, nicotine [32]. These results suggest that in PC12 cells, ERK activation is required for NGF-stimulated neurite outgrowth [27,28]. In this study, the percentage of neurite-bearing cells decreased after exposure to IH4 in NGF-stimulated PC12 cells. The lower percentage of differentiated cells induced by IH was abolished by the ROS scavengers SOD and Phe, PP2A inhibitors, OKA and Can and the ERK phosphorylation inducer nicotine. These results suggest that IH-induced ROS generation plays a role in inhibiting NGF-induced PC12 differentiation, which results from PP2A activation and downregulated ERK1/2 activation.

Notably, the frequency of IH in clinical settings far exceeds that of sustained chronic hypoxia, which typically occurs during high-altitude sojourns. Sustained exposure to IH, in the absence of significant sleep deprivation, induces substantial neurocognitive impairments in both adult and developing rodents [5]. Further, apnoea of prematurity (AOP) has a higher incidence in preterm infants because of disturbed breathing control, which leads to apnoea and IH. The frequency and severity of AOP has been linked to adverse outcomes, including abnormal myelination, synaptic connections and mental development [33].

Neuronal development in the adult hippocampus involves three levels of proliferating cells, presumably stem or progenitor cells, and from a progenitor cell stage well into a stage of postmitotic differentiation [34]. These functional alterations are accompanied by evidence of increased oxidative stress, induction and propagation of inflammatory processes, and consequent neuron cell losses via the induction of apoptotic mechanisms in selected brain regions, such as the frontal cortex and the CA1 region of the hippocampus [5]. Therefore, our findings provide a rationale for future research to develop better therapeutic strategies for patients with sleep breathing disorders to prevent memory and learning impairments.

## Conclusion

**Figure 6:**
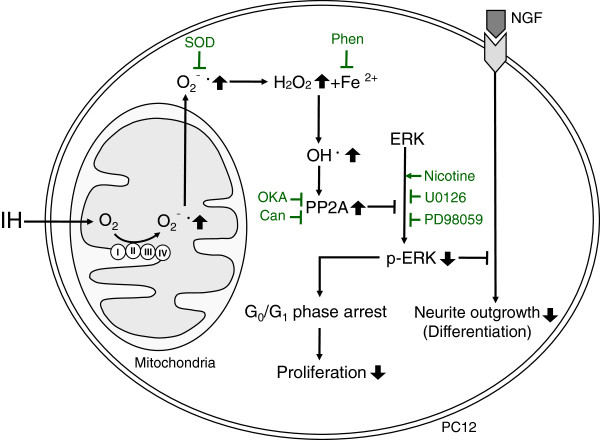
**Possible molecular mechanisms for intermittent hypoxia (IH)-induced activation of protein phosphatase 2A (PP2A) and inhibition of extracellular signal-regulated kinase 1/2 (ERK1/2) phosphorylation contribute to inhibiting PC12 cell proliferation through G**_**0**_**/G**_**1 **_**phase arrest and nerve growth factor (NGF)-induced neuronal differentiation.**

Our study results suggest that IH-induced ROS generation increases PP2A activation and subsequently inhibits ERK1/2 activation, which leads to inhibiting cell proliferation through G_0_/G_1_ phase arrest and NGF-induced neuronal differentiation of PC12 cells (Figure 6).

## Abbreviations

PP2A: Protein phosphatase 2A; IH: Intermittent hypoxia; RA: Room air; PC12: Pheochromocytoma-12 cell; ROS: Reactive oxygen species; ERK1/2: Extracellular signal-regulated kinase 1/2; NGF: Nerve growth factor; SOD: Superoxide dismutase; MnTBAP: Mn(III)tetrakis(4-benzoic acid)porphyrin (MnTBAP) chloride; Phe: 1,10-phenanthroline; OKA: Okadaic acid; Can: Cantharidin; AOP: Apnoea of prematurity.

## Competing interests

The authors declare that there are no competing interests.

## Authors’ contributions

Conceived and designed the experiments: TIC, KTY. Performed the experiment: CHW, PYC, HST, LJH. Contributed reagents/materials/analysis tools: TIC, KTY. Analyzed the data: TIC, CHW, HST, LJH. Wrote the paper: TIC, KTY. All authors read and approved the final manuscript.
